# Peculiarities of Synthesis and Properties of Lignin–Silica Nanocomposites Prepared by Sol-Gel Method

**DOI:** 10.3390/nano8110950

**Published:** 2018-11-18

**Authors:** Tetyana M. Budnyak, Selda Aminzadeh, Ievgen V. Pylypchuk, Anastasia V. Riazanova, Valentin A. Tertykh, Mikael E. Lindström, Olena Sevastyanova

**Affiliations:** 1 Division of Wood Chemistry and Pulp Technology, Department of Fiber and Polymer Technology, KTH Royal Institute of Technology, Teknikringen 56-58, SE-100 44 Stockholm, Sweden; mil@kth.se; 2 Chuiko Institute of Surface Chemistry, National Academy of Sciences of Ukraine, 17 General Naumov Str., 03164 Kyiv, Ukraine; tertykh@yahoo.com; 3 Wallenberg Wood Science Center (WWSC), Department of Fiber and Polymer Technology, KTH Royal Institute of Technology, Teknikringen 56-58, SE-100 44 Stockholm, Sweden; seldaa@kth.se (S.A.); anaria@kth.se (A.V.R.); 4 Department of Molecular Sciences, Swedish University of Agricultural Sciences (SLU), Allmas alle 5, SE-750 07 Uppsala, Sweden; ievgenpylypchuk@gmail.com

**Keywords:** lignin, silica, sol-gel process, hybrid composites, thermal analysis

## Abstract

The development of advanced hybrid materials based on polymers from biorenewable sources and mineral nanoparticles is currently of high importance. In this paper, we applied softwood kraft lignins for the synthesis of lignin/SiO_2_ nanostructured composites. We described the peculiarities of composites formation in the sol-gel process through the incorporation of the lignin into a silica network during the hydrolysis of tetraethoxysilane (TEOS). The initial activation of lignins was achieved by means of a Mannich reaction with 3-aminopropyltriethoxysilane (APTES). In the study, we present a detailed investigation of the physicochemical characteristics of initial kraft lignins and modified lignins on each step of the synthesis. Thus, 2D-NMR, ^31^P-NMR, size-exclusion chromatography (SEC) and dynamic light scattering (DLS) were applied to analyze the characteristics of pristine lignins and lignins in dioxan:water solutions. X-Ray photoelectron spectroscopy (XPS) and Fourier transform infrared (FTIR) were used to confirm the formation of the lignin–silica network and characterize the surface and bulk structures of the obtained hybrids. Termogravimetric analysis (TGA) in nitrogen and air atmosphere were applied to a detailed investigation of the thermal properties of pristine lignins and lignins on each step of modification. SEM confirmed the nanostructure of the obtained composites. As was demonstrated, the activation of lignin is crucial for the sol-gel formation of a silica network in order to create novel hybrid materials from lignins and alkoxysilanes (e.g., TEOS). It was concluded that the structure of the lignin had an impact on its reactivity during the activation reaction, and consequently affected the properties of the final hybrid materials.

## 1. Introduction

The development of advanced materials from biorenewable sources is currently of high importance. Lignin, an aromatic biological macromolecule, has great potential for replacing oil-based polymers. As a cross-linked natural phenolic polymer, lignin is of great interest because of its valuable properties and abundance as a by-product in the pulp and paper industries [1]. The chemical structure of kraft lignin, a by-product of sulfate pulping, in terms of the molecular weight (Mw), content of functional hydroxyl and carboxyl groups, and cross-linking density, differs significantly from that of lignin in the original plants as a result of the alkaline treatment [2,3,4]. Kraft lignin is used today almost exclusively for energy production; however, there has been growing interest in the extraction of a portion of kraft lignin from black liquor for use in high-value applications in parallel to pulp production [5,6,7]. Often, additional purification and fractionation treatments of kraft lignin are needed prior to its use in polymeric applications. One such method is the LignoBoost process, where kraft lignin is purified in sequential precipitation and filtration steps [8]. Other alternatives to obtaining lignin materials of high purity with a suitable molecular weight and polydispersity are the ultrafiltration of black liquor with a ceramic membranes or solvent fractionation of lignin [9,10,11,12,13,14,15,16,17,18]. There have been several reports in the literature on methods of lignin modification or graft polymerization to obtain lignin-based composites, epoxy resins, biomedical materials, carbon fibers, etc. [19,20,21,22,23,24,25,26,27,28].

There is growing interest in the development of advanced materials based on a combination of biomacromolecules and inorganic carriers to form organic–inorganic hybrid materials [29]. In the case of technical lignins, the preliminary activation of the biopolymer is often required to improve the reactivity [19]. A silica/lignin composite was obtained in the process of silica surface modification with oxidized lignin solution [30,31,32,33,34], by grafting additional functional groups or polymerizable moieties to lignin [35], by the simultaneous mechanical mixing of the initial silica and kraft lignin powders [36,37], or by direct precipitation in aqueous solution [38]. The obtained composites were found to be efficient as polymer fillers [37], electrochemical sensors [33], and biosorbents for the removal of toxicants from aqueous solutions [35,39,40,41].

Among the different techniques for obtaining hybrid materials, sol-gel technology offers a simple and convenient method for preparing organic–inorganic composite materials on a molecular level [42]. In the literature, there is a limited number of reports on obtaining lignin–silica nanomaterials by the sol-gel method. A paper by Qu et al. [43] reported the preparation of lignin–silica hybrid in situ using the sol-gel process. The authors investigated the optimum conditions for the hybrid formation and its pore structure. It was suggested that such material could be used for the decontamination of environmental objects. In other works [44,45], authors applied a modification of hydrolyzed lignin with silica oligomers to prepare hybrids via the sol-gel process. However, neither of these works presented a controlled synthesis of homogeneous lignin–silica composites, but rather used a mixture of organic and inorganic components.

The main problem in the application of kraft lignin as a component of hybrid composites is the complexity of its chemical modification. On the one hand, kraft lignin is a highly hydrophobic material that is difficult to involve in reactions that require aqueous media, such as the sol-gel reaction. On the other hand, the chemical modification of lignins by reactive groups, which are capable of cohydrolyzing with common sol-gel reagents (e.g., tetraethoxysilane (TEOS)), is not an easy task. Therefore, our goal was to chemically modify lignin in order to graft functional groups into lignin macromolecules that are capable of cohydrolyzing with TEOS (sol-gel reaction) in water-miscible solvent. The physicochemical properties of initial, activated, and immobilized lignins into final hybrid materials were investigated and compared by means of size-exclusion chromatography (SEC), nuclear magnetic resonance (NMR), dynamic light scattering (DLS), X-Ray photoelectron spectroscopy (XPS), Fourier transform infrared (FTIR), thermogravimetric analysis (TGA), scanning electron microscopy (SEM), and the adsorption method.

## 2. Materials and Methods

### 2.1. Materials

The LignoBoost Kraft lignin (LBL) samples that were used in this study were produced from Nordic softwood and kindly supplied by a plant in Northern Europe. The low-Mw lignin (CFBL) that was used in this study was produced with an ultrafiltration unit equipped with a ceramic membrane with a molecular weight cut-off of 5 kDa that was kindly provided by the Clean Flow Black company (Forshaga, Sweden). Tetraethoxysilane (TEOS), 3-aminopropyltriethoxysilane (APTES), 1,4-dioxane, and formaldehyde were purchased from Sigma-Aldrich (St. Louis, MO, USA). Ethanol was obtained from VWR. All the chemicals were of reagent grade.

### 2.2. Methods

Synthesis of the silica. TEOS hydrolysis was carried out in a mixture of dioxane/water (mass ratio 4:1) or distilled water. The reaction mixture was stirred for 3 h in the acidic medium to form the sol. After 24 h, the obtained silica was vacuum-dried at 25 °C overnight and washed twice by ethanol and water.

Chemical activation of lignin. The lignin–Mannich compounds were synthesized as a pre-modification step of LBL or CFBL by with APTES in the presence of formaldehyde.

Synthesis of lignin–silica hybrid composites and lignin–Mannich compounds. Lignin–silica composites were obtained by the sol-gel method. For that, TEOS was hydrolyzed in a pre-modified by Mannich reaction lignin. The detailed procedure was presented in Budnyak et al. [46].

### 2.3. Analysis

#### 2.3.1. Size-Exclusion Chromatography (SEC)

The molecular weight of the lignin samples was characterized by size-exclusion chromatography using an SEC 1260 Infinity instrument (Polymer Standard Services, Mainz, Germany) coupled to a dual system detector (UV, RI). DMSO + 0.5% LiBr (*w*/*w*) was used as the mobile phase. The separation system consisted of a PSS GRAM Precolumn, PSS GRAM 100 Å, and PSS GRAM 10,000 Å analytical columns thermostated at 60 °C and connected in series. Pullulan standards with Mw within the range 708 kDa, 337 kDa, 194 kDa, 47.1 kDa, 21.1 kDa, 9.6 kDa, 6.1 kDa, 1.081 kDa, and 342 Da were used for the standard calibration.

#### 2.3.2. Nuclear Magnetic Resonance Spectroscopy (NMR)

##### ^31^P-NMR

The content of functional groups in lignin samples was measured by ^31^P NMR [47]. Approximately 20–30 mg of lignin samples was weighed and phosphitylated using 2-chloro-4,4,5,5-tetramethyl-1,3,2-dioxaphospholane. Endo-*N*-hydroxy-5-norbornene-2,3-dicarboximide (e-HNDI) (Sigma Aldrich, 40 mg/mL) and chromium (Ш) acetylacetonate (Aldrich, 5 mg/mL) were used as an internal standard and a relaxation reagent, respectively. The derivatized sample was dissolved in CDCl_3_ prior to analysis. The ^31^P NMR experiment was performed with a 90° pulse angle, inverse gated proton decoupling, and a delay time of 10 s. For analysis, 256 scans with a time delay of 6 s and a total runtime of 34 minutes were collected. Measurements were performed in duplicate.

##### 2D-Heteronuclear Single Quantum Coherence (2D-HSQC) NMR

Approximately 100 mg of sample was acetylated for better solubility [48]. The residue was dissolved in 700 µL of DMSO-*_d_*_6_. The two-dimensional (2D) HSQC NMR spectrum was acquired using the Bruker, Billerica, MA, USA pulse program ‘hsqcetgpsi’ a relaxation delay of 1.7 s, a coupling constant of 145 Hz, an INEPT transfer delay time of 1.72 ms (*d*4 = 1/4 J), a spectral window of 10.5 ppm in *F*2, and 166 ppm in *F*1 with 1024 × 512 increments, 240 scans per increment, and a spectral center set at 90.0 ppm in *F*1 and 5.3 ppm in *F*2. The two-dimensional (2D) NMR dataset was processed with 2 K × 1 K data points using a π/2-shifted sine-bell window function in both dimensions. Central DMSO (δ_C_/δ_H_ = 39.5/2.5 ppm) was used as an internal reference according to the adopted solvent.

##### Dynamic Light Scattering (DLS)

The particle size of the LignoBoost lignin and CleanFlowBlack lignin was measured using a dynamic light scattering meter (Zetasizer, Nano ZS, Malvern Instruments, Malvern, England). For the measurements, solutions of lignin with a concentration of 1 g·L^−1^ were used. A dioxane/water mixture with a volume ratio of 4:1 was used as the solvent.

##### FTIR Spectroscopy

IR spectra were collected using a Perkin-Elmer spectrophotometer (Spotlight 400 FTIR imaging system, Waltham, MA, USA) equipped with a Spectac MKII Golden Gate system (Creekstone Ridge, GA, USA). The samples were analyzed in the range of 600–4000 cm^−1^ with 16 scans at a four cm^−1^ resolution and a one cm^−1^ interval at room temperature.

##### X-Ray Photoelectron Spectroscopy (XPS)

XPS spectra were recorded on an ESCA apparatus with a multidetection electron analyzer (Scienta R4000, produced by VG Scienta, East Sussex, UK) in fixed analyzer transmission mode.

##### Thermal Analysis

Thermal analysis was carried out on a TGA/DSC 1 (Mettler Toledo, Columbus, OH, USA) instrument under the following operational conditions: a heating rate of 10 °C min^−1^, a dynamic atmosphere of synthetic air or nitrogen (50 mL·min^−1^), a temperature range of 30–900 °C, and a sample mass of ~2.5 mg.

##### Scanning Electron Microscopy (SEM)

The structural characteristics of the fabricated samples were studied with a field-emission scanning electron microscope (FE-SEM, S-4800, Hitachi, Japan). The SEM images were obtained using a secondary electron detector. The samples were coated with a 1-nm thick Pt–Pd layer sputtered with a Cressington (Watford, UK), 208HR high-resolution coater.

## 3. Results and Discussion

### 3.1. Characterization of the Technical Lignins

**Figure 1:**
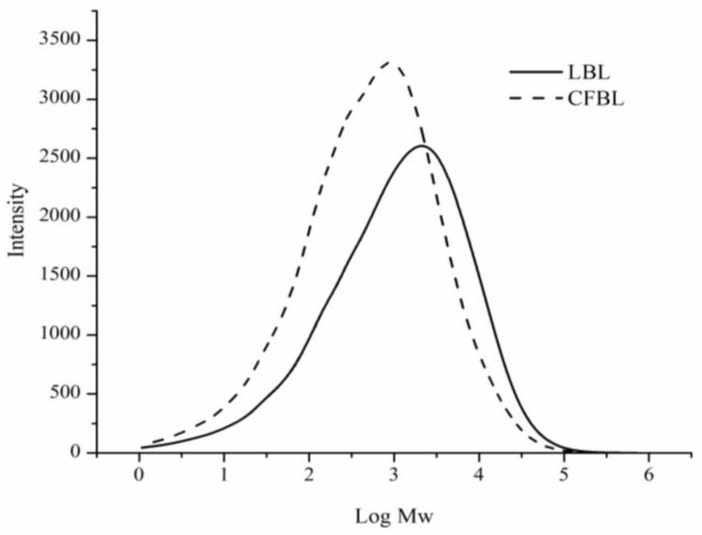
Molecular weight for LBL and CFBL based on the results obtained from SEC.

The molecular weights of LBL and CFBL, measured by the SEC method, were approximately 5600 and 3000 Da, respectively (Table 1). The polydispersity index (PDI) was higher for the LBL lignin sample than for the CFB (see Table 1). The molecular weight distribution curves are shown in Figure 1. For both lignin samples, one major peak can be observed in chromatogram; however, a shift toward a higher molecular weight can be clearly seen for the LBL lignin.

**Figure 2:**
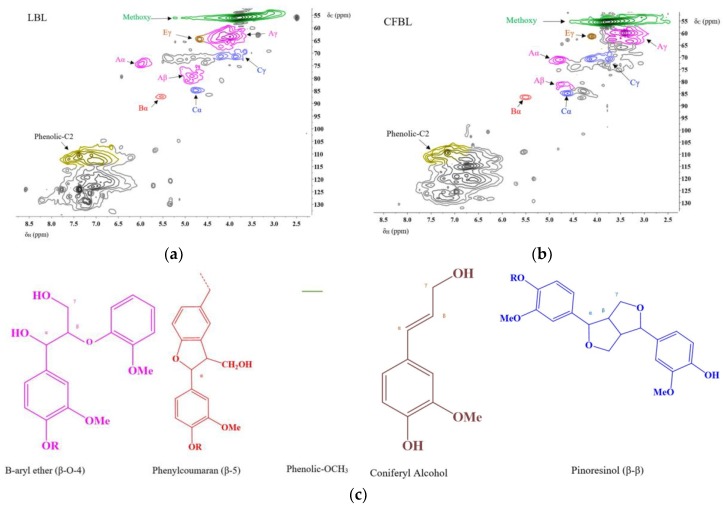
Interunit analysis by 2D-NMR for the: (**a**) LBL and (**b**) CFBL lignin samples; (**c**) assignments.

The various types of interunit linkages for lignin samples were investigated and semiquantified by the 2D-HSQC NMR method (Figure 2). The peak assignment was performed based on the works by Capanema et al. Del Río et al. Kim and Ralph, Zhang and Gellerstedt [49,50,51,52]. The signal from the phenolic carbon-2 was used as an internal standard to quantify different linkages. As illustrated in the 2D NMR spectra of LBL and CFBL (Figure 2a,b), both lignins contain the typical for lignin signals from the β-aryl ether (β-O-4), pinoresinol (β-β), phenylcoumarin (β-5), with the coniferyl alcohol structures and the methoxy groups in similar proportions (Figure 2c).

The ^31^P NMR method was used to quantify the amount of various functional groups in both lignin samples (Table 1, Figure S1). Table 1 shows the total content of functional phenolic and hydroxyl groups in both lignin samples. CFBL had a higher content of phenolic and aliphatic hydroxyl groups than LBL. As reported in our previous work, low Mw fractions of lignin are usually enriched in phenolic compounds [10], indicating the possibility of higher reactivity for such lignin samples. Based on the quantification, the amount of total non-condensed structures for CFBL lignin with a lower molecular weight, which was obtained by the ultrafiltration of black liquor, was 21%, and that for LBL, which was obtained by the sequential precipitation of black liquor and had a higher molecular weight, was 16%. CFBL had also a higher ratio of non-condensed to condensed aromatic units than LBL (1.59 versus 1.12), which indicates the presence of a less condensed structure and possibly the higher reactivity of the CFBL lignin.

### 3.2. Dynamic Light Scattering

**Figure 3:**
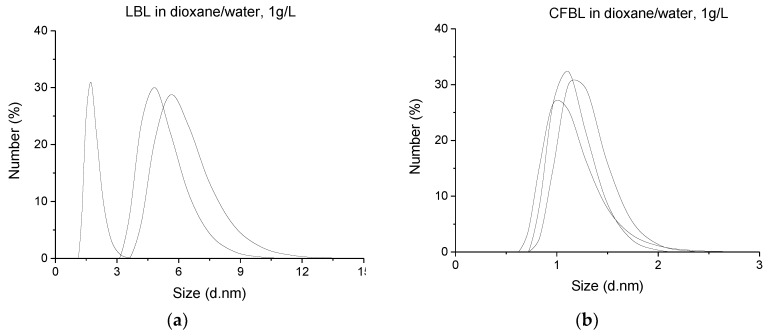
Dynamic light scattering (DLS) diagrams of the particle size distributions of: (**a**) LBL and (**b**) CFBL in a mixture of dioxane/water (4:1).

Dynamic light scattering was applied to investigate the size distribution of lignin macromolecules in a dioxane/water mixture with a ratio of 4:1, as this solvent was used for the synthesis. The plots of the Z-average size distributions by number of the LBL and CFBL macromolecules are presented in Figure 3. According to the Z-average data, the hydrodynamic diameter of the lignin’s particles (or micelles) range in the chosen solvent mixture was 1.3–12 nm for LBL and 0.7–2.4 nm for CFBL, which is in good agreement with the existing literature data [53,54].

In organic solvents, lignin has a swollen, extended structure. Due to their hydrophobic nature, lignin molecules fold when in contact with water. In our opinion, the immobilization (distribution) of lignin molecules in a silica network would prevent the folding of the molecules upon contact with water; thus, better accessibility of the functional groups can be achieved.

### 3.3. Design and Synthesis of Sorbent

**Scheme 1:**
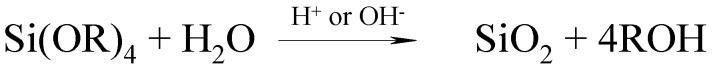
General scheme for the synthesis of silica by the sol-gel reaction.

The sol-gel method has proven to be useful for generating polymer–silica hybrid materials. The methods of modification of a silica surface with phenol-containing compounds by the Mannich reaction was developed earlier in the works of Tertykh et al. and Yanovska et al. [55,56]. Conducting the in situ formation of silica in the presence of a solution containing macromolecules facilitates the formation of hybrid materials with covalent bonds between the organic and inorganic phases [42,57,58]. However, the sol-gel reaction requires the addition of water (Scheme 1).

**Scheme 2:**
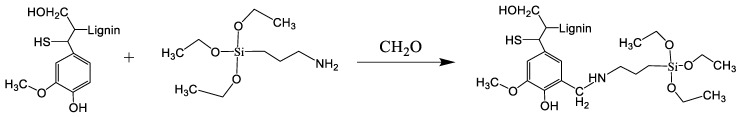
Activation of lignin with 3-aminopropyltriethoxysilane (Mannich reaction).

Incorporating pristine (nonactivated) lignin into the sol-gel process resulted in a heterogeneous mixture of silica and lignin, where lignin was easily removed by washing with dioxane. To improve the reactivity of the lignin samples, which are not capable of undergoing a polycondensation reaction with TEOS, the lignin was activated by an aminomethylation (Mannich) reaction with APTES. Due to the presence of active protons in the lignin structure, formaldehyde forms a methylene bridge between APTES and lignin (Scheme 2).

**Scheme 3:**
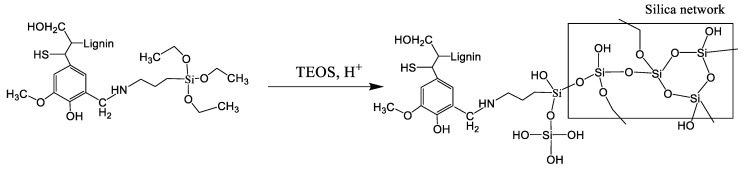
Hydrolysis of tetraethoxysilane (TEOS) in the presence of modified lignin (sol-gel reaction).

The modification of lignin by the Mannich reaction is a particular type of amination process where typically one dialkylaminomethyl group is introduced at the *ortho* position of a free phenolic hydroxyl group through a reaction with formaldehyde and amine [59]. According to the study of Du et al. [60], there is the primary product, the so-called Mannich condensation product, where the nitrogen-containing group is introduced at the C_5_ position of the benzene ring; however, some amount of side reactions could take place [61]. To eliminate the risk of involving side products in the second stage of the synthesis of hybrid material, the product of the Mannich reaction was precipitated and washed by diethyl ether. In the Supplementary Materials (Figure S2), the FTIR spectra of lignin before and after washing by diethyl ether are presented. It can be seen that a characteristic band at 1738 cm^−1^, which corresponds to the presence of side products (preferably imines), completely disappeared after washing with ether. Thus, activated lignin washed with diethyl ether was cohydrolyzed with TEOS in an acidic medium (Scheme 3). According to the well-established sol-gel reaction, a silica network was formed.

### 3.4. FTIR Analysis

**Figure 4:**
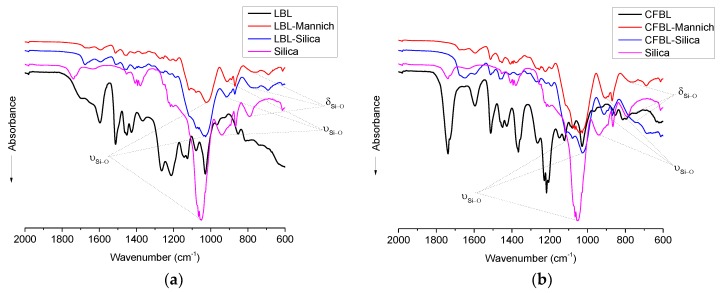
Fourier transform infrared (FTIR) spectra of the materials based on (**a**) LBL and (**b**) CFBL: initial lignin; lignin modified by the Mannich reaction; lignin-silica hybrids and synthesized pure silica.

To confirm the formation of silica as part of the hybrid lignin–silica composites, the FTIR spectra of the initial and modified lignins and synthesized composites were compared (Figure 4a,b). In the FTIR spectra of LBL and CFBL, the band at 3412 cm^−1^ corresponds to the O–H stretching vibrations O–H in the hydroxyl groups bound to carbon atoms. The intense absorption bands at 2800–3000 cm^−1^ were assigned to the C–H stretching vibrations of the methyl and methylene groups of lignin. Bands at 1600 cm^−1^, 1230 cm^−1^, and 1500 cm^−1^ corresponding to aromatic skeletal vibrations plus C=O stretching, C–C, C–O, and C=O stretching, and the aromatic skeleton, were observed in all of the lignin-containing samples. In lignin samples, the band at 1030 cm^−1^ was assigned to aromatic C–H in-plane deformation and the deformation vibrations of the –C–O bonds in the primary alcohols. In lignin–Mannich and lignin–silica synthesized silica samples, an intense absorbance at 1068 cm^−1^ and 950 cm^−1^ represent Si–O stretching vibrations in Si–O–Si; and at 795 cm^−1^, –Si–O deformational vibrations, confirming the formation of a silica network.

### 3.5. XPS Analysis

XPS was used to study the hybrid lignin–silica composite surface structure. This method is based on changes in the electronic states of the elements. Moreover, XPS analysis could be used to confirm the successful aminomethylation of lignin and the formation of a silica network during the sol-gel reaction.

**Figure 5:**
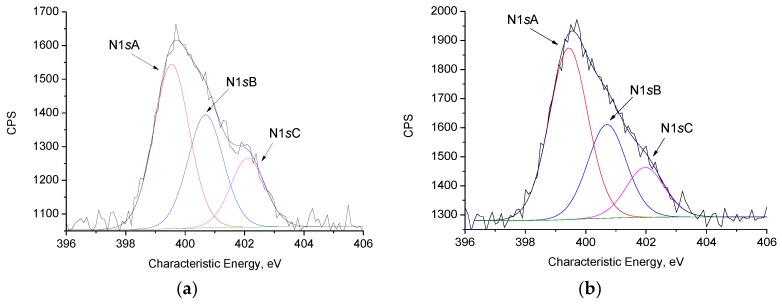

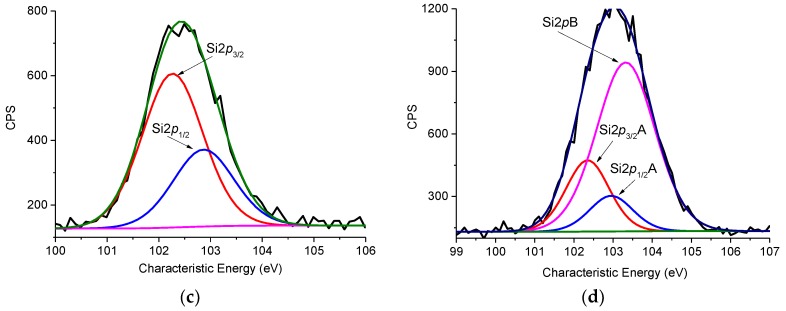
X-Ray Photoelectron Spectroscopy (XPS) spectra of the N1*s* (**a**,**b**) and Si2*p* (**c**,**d**) levels for the CFBL–Mannich (**a**,**c**) and CFBL–silica (**b**,**d**) samples.

The original and deconvoluted XPS spectra of CFBL modified by the Mannich reaction (CFBL–Mannich), and the corresponding lignin–silica (CFBL-Silica) hybrid are presented in Figure 5a,b, respectively. The N1*s* spectra of the CFBL–Mannich and CFBL–silica samples are represented by three nonequivalent nitrogen atoms. N1*s* with binding energy *E*_bind_ = 399.5 eV can be assigned to the secondary amino groups of APTES radical (−CH_2_NHCH_2_) as well as to the −C−N− bond between APTES and lignin residues after the Mannich reaction. Nitrogen with a binding energy *E*_bind_ = 400.7 eV could be related to the nitrogen in the −C=N bond after an APTES amino group reaction with formaldehyde (possible azomethine bond formation). The N with *E*_bind_ = 402.1 eV corresponds to the quaternary N, which was possibly obtained after amino group protonation (R_2_NH^+^). Compared with a CFBL–Mannich composite, the ratio between the −C−N− and −C=N bonds in CFBL-silica changes in favor of the −C−N− of the CFBL–silica composite. The N/Si ratio for the CFBL–Mannich composite is 0.614 and decreased up to 0.307 for the CFBL–silica composite.

As can be observed from the Si2*p* XPS spectra presented in Figure 5c, there are two types of silicon atoms in the CFBL–Mannich composite. The Si2*p*_3/2_ band with a binding energy of 102.3 eV can be assigned to the silicon in siloxane bonds as well as the unreacted Si(OEt)_3_ residue of APTES or TEOS. After the addition of TEOS to the CFBL–Mannich composite (Figure 5d), a third, nonequivalent Si electronic state at *E*_bind_ = 103.3 eV can be observed. This state can be related to the SiO_2_ being formed after the introduction of TEOS and polycondensation (Table 2).

According to the atomic concentrations (%) of Si and C atoms in the activated lignin (CFBL–Mannich sample), one atom of Si is incorporated per 12 atoms of C (C:Si = 9:0.75). This result led us to conclude that almost every C9 phenolic unit of lignin reacted with an aminosilane (APTES) molecule. In the case of the CFBL–silica hybrid, the ratio of carbon to silicon changes to 6:1.

### 3.6. Morphology and Textural Characteristics

**Figure 6:**
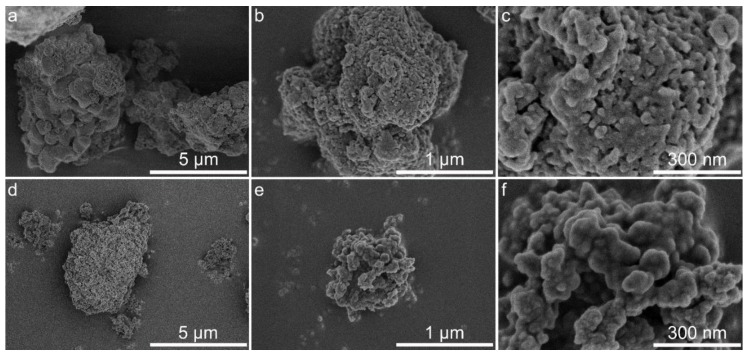
SEM images of the original LignoBoost lignin (**a**–**c**) and the LignoBoost lignin–silica hybrid (**d**–**f**) (high-resolution topographic contrast secondary electron imaging, parameters: accelerating voltage = 1 kV; working distance: (**a**–**c**) 1.4 mm, (**d**–**f**) 1.8 mm; magnification: (**a**,**d**) ×10,000, (**b**,**e**) ×50,000, (**c**,**f**) ×150,000).

**Figure 7:**
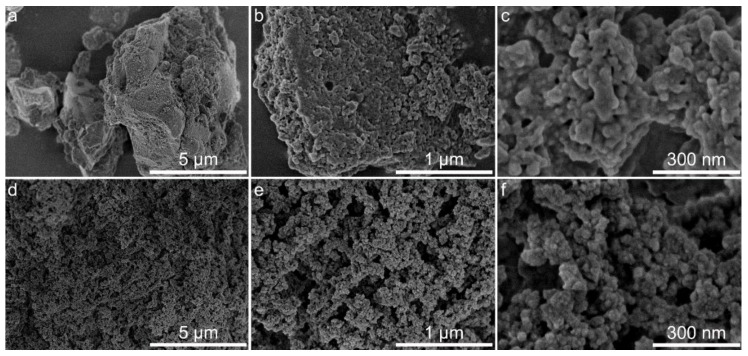
SEM images of the original CleanFlowBlack lignin (**a**–**c**) and the CleanFlowBlack lignin–silica hybrid (**d**–**f**) (high-resolution topographic contrast secondary electron imaging, parameters: accelerating voltage = 1 kV; working distance: (**a**–**c**) 1.4 mm, (**d**–**f**) 1.7 mm; magnification: (**a**,**d**) ×10,000, (**b**,**e**) ×50,000, (**c**,**f**) ×150,000).

**Figure 8:**
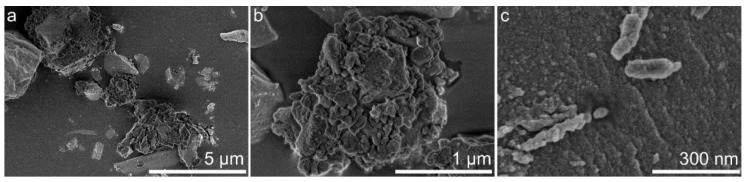
SEM images of synthesized silica (high-resolution topographic contrast secondary electron imaging, parameters: accelerating voltage = 1 kV; working distance = 1.4 mm; magnification: (**a**) ×10,000, (**b**) ×50,000, (**c**) ×150,000).

The morphologies of the initial lignins and obtained hybrid materials were characterized via SEM analysis. Figure 6, Figure 7 and Figure 8 present the structures of the original and silica-containing lignin (Figure 6a–f, respectively), CFBL lignin (Figure 7a–f, respectively), and reference silica particles (Figure 8).

As seen from Figure 6a–c and Figure 7a–c, the structures of the original lignins are rather dense, and most of the nanoparticles are in close contact with each other. After modification (Figure 6d–f and Figure 7d–f), the morphologies of the lignin–silica hybrid composites become rougher and more friable, which is somewhat typical for hybrid materials obtained by the sol-gel method. This observation presumably confirms the creation of a silica network between the particles of lignin. Additionally, the formation of a rough and irregular surface with plenty of holes, canals, and cavities most likely causes a desirable increase in the specific surface area of the fabricated hybrid materials. The nanosized structure of the hybrids’ surface could be observed in Figure 6f and Figure 7f.

According to the data from the nitrogen adsorption/desorption isotherms presented in Budnyak et al. [46], the LBL–silica and CFBL–silica composites have a Brunauer–Emmett–Teller (BET) specific surface areas of 74 m^2^·g^−1^ and 92 m^2^·g^−1^, respectively, and belong to mesoporous materials with some amount of micropores. A detailed investigation of the textural characteristics of lignin–silica hybrids and initial polymers is presented in Budnyak et al. [46]. By comparing the data for the original lignins with those of the hybrid composites, it is evident that the creation of a silica network in the presence of lignin results in an increase in porosity, which causes an increment of the specific surface area of the obtained materials. However, the specific surface area and porosity of the obtained hybrids are relatively low in comparison with those of the inorganic materials or hybrids with a high content of the inorganic component, [42,58,62] which is in agreement with the data obtained by other research groups [20,63,64].

### 3.7. Thermal Analysis

**Figure 9:**
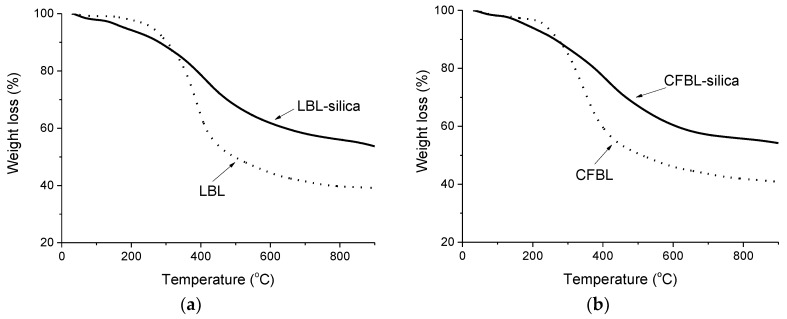

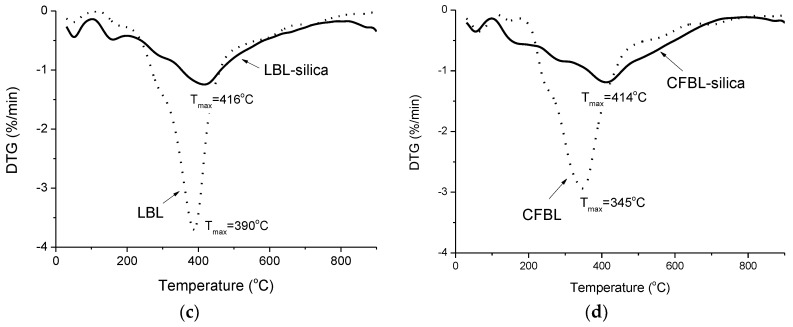
TG (**a**,**b**) and DTG (**c**,**d**) curves of thermal decomposition in a N_2_ atmosphere for the original LBL and CFBL lignins.

TGA was performed to characterize the thermal properties of the original lignins and synthesized hybrids. The destruction processes of the initial LBL and CFBL lignins and the hybrid composites under a N_2_ atmosphere are presented in Figure 9. The obtained TG and DTG curves have similar thermal behaviors; however, there are some significant differences in the thermal characteristics of the two types of original technical lignins compared to the immobilized ones. At the first stage of thermal decomposition of the initial LBL and CFBL, gradual moisture evaporation and water evaporation due to self-condensation reactions (peaks at 51 °C and 184 °C, 62 °C and 150 °C, respectively) occur. The maximum temperatures for the conversion of phenols into pyrocatechols [65] and the conversion of short substituents of the benzene rings [66] were found at 390 °C and 345 °C for the original LBL and CFBL, respectively. The rearrangement of the backbone and carbonization of the studied lignins takes place at temperatures above 400 °C.

It was found that the maximum temperature of the polymer destruction decreased after each stage of lignin modification. Thus, for activated lignins by Mannich reaction (samples LBL–Mannich and CFBL–Mannich), the maximum for condensation and elimination of the –OH groups and conversion of phenols into pyrocatechols occurs at 338 °C and 333 °C, respectively. In the case of lignin–silica composites, the maximum for this processes was found at 290 °C for both samples. The maximum temperature of the conversion of short substituents of the benzene rings, the rearrangement of the backbone, and carbonization of LBL–Mannich and CFBL–Mannich was detected at 464 °C and 422 °C, respectively, and for LBL-based and CFBL-based composites, the maximum shifted to 416 °C and 414 °C. The concentrations of moisture in both lignin–silica composites are below 1% (Figure 9b). The total weight loss for the LBL–silica and CFBL–silica composites was 45% and 46%, respectively, and for the original LBL and CFBL samples, it was 61% and 59%, respectively. The main destruction regions are presented in Table 3.

The characteristics of the thermal decomposition of studied materials in O_2_ atmosphere are presented in Table S1 (Supporting Information). TG and DTG curves of thermal decomposition in N_2_ and O_2_ atmosphere for initial and modified lignins, hybrids, and silica presented in Figures S4 and S5 of the Supporting Information, respectively.

### 3.8. Application as Sorbzents in Water Treatment

Synthesized hybrid materials were found to be effective as adsorbents for organic pollutants from aqueous solutions. Figure S6 presents a comparison of the adsorption capacity of the hybrids, initial lignins, and silica toward methylene blue dye. The interesting observations are that the adsorption activity of the lignins is improved, and that the difference in the lignin structure affects the performance of the lignin to a more substantial degree as a result of immobilization on a silica surface. Our detailed study of the adsorption activity of synthesized materials for the removal of methylene blue dye from aqueous solutions was presented in previous work [46]. In the study, the effects of parameters such as the contact time, initial concentration of dye, and initial pH on the adsorption capacity was evaluated. Thus, the lignin–silica composites were proved to be potential materials for application in wastewater treatment.

## 4. Conclusions

The proposed method for lignin modification using APTES according to the Mannich reaction was found to be an effective way to activate kraft lignin. As demonstrated in the current study, the activation of lignin is necessary for the sol-gel formation of a silica network to create novel hybrid materials from lignins and alkoxysilanes (e.g., TEOS). According to the XPS data, the carbon:silicon ratio in the activated lignin was found to be 12:1 (9:0.75), which led us to conclude that almost every C9 phenolic unit of lignin reacted with APTES. In the case of the CFBL–silica hybrid, the ratio of carbon to silicon changed to 6:1. A comparison of the FTIR spectra of the initial and modified lignins and the synthesized composites confirmed the creation of a silica network as a part of the hybrid lignin–silica composites.

The proposed method allowed us to obtain porous hybrid materials with an increased surface area and a somewhat rough and friable structure with plenty of holes, canals, and cavities. It was found that a silica network was created between the lignin particles. This structure was confirmed by SEM analysis, where it was observed that in the case of the hybrid materials, the nanoparticles of lignin were better separated from each other. The fact that the modification of lignin with alkoxysilanes (APTES and TEOS) promotes the formation of cross-linked lignin particles interconnected by an inorganic matrix was also confirmed by the textural characteristics.

It was concluded that the structure of the lignin had an impact on its reactivity during the activation reaction, and consequently affected the properties of the final hybrid materials. Thus, the thermal stability of the immobilized lignin increased in comparison to the original lignins: the maximum destruction temperature of the original CFBL and LBL samples shifted from 345 °C to 414 °C and from 390 °C to 416 °C, respectively, after their immobilization in the hybrid composites. It was demonstrated that the synthesized hybrid materials could be used as effective sorbents for organic molecules from aqueous solutions.

## Figures and Tables

**Table 1:** Contents of functional groups in LBL and CFBL lignins measured by ^31^P-NMR. MW: molecular weight.

Sample	Molecular Weight			Content of Functional Groups, mmol·g^−1^					
	Mw, Da	Mn, Da	PDI	Phenolic OH			Aliphatic OH	Total OH	COOH
				Condensed	Non-Condensed	Total			
LBL	1300	5600	4.2	1.88	2.11	3.99	1.73	5.72	0.41
CFBL	880	3000	3.5	2.11	3.36	5.47	1.81	7.28	0.28

**Table 2:** XPS data of the Si2*p* levels for the CFBL–silica and CFBL–Mannich samples.

Sample	Atom	Position	Concentration, at.%	Assignment
CFBL–silica	N1*s*A	399.4	54.4	amine, amide, cyanides
	N1*s*B	400.7	29.7	imide
	N1*s*C	402.0	15.8	quaternary nitrogen
	Si2*p*_3/2_A	102.3	32.1	silicon/siloxane (SiOEt)_3_ from TEOS and 3 aminopropyltriethoxysilane (APTES) (unhydrolyzed ethoxy groups)
	Si2*p*B	103.3	67.9	SiO_2_
CFBL–Mannich	N1*s*A	399.5	47.8	amine, amide, cyanides
	N1*s*B	400.7	32.8	imide
	N1*s*C	402.1	19.4	quaternary nitrogen
	Si2*p*_3/2_	102.3	100	silicon/siloxane, (SiOEt)_3_

**Table 3:** Characteristics of thermal decomposition in the N_2_ atmosphere of studied materials.

M.	T_max_, °C (DTG)	∆m, % (TG)	∆m_total_, % (TG)	Process
LBL	51	<0.4	61	Moisture evaporation
	184			Water evaporation due to self-condensation reactions (up to 400 °C) [66]
	293			The β–β and C–C linkages between the lignin monomeric units cleave at 275–350 °C, while the recombination of the formed radicals leads to guaiacyl and syringyl compounds [67]; aryl-ether bonds cleavage [68]
	390			Conversion of phenols into pyrocatechols [65], the conversion of short substituents of the benzene rings [66]
	≥400			Rearrangement of backbone, carbonization
CFBL	62	<0.4	59	Moisture evaporation
	150			Water evaporation due to self-condensation reactions (up to 400 °C) [66]
	256			The β–β and C–C linkages between the lignin monomeric units cleave at 275–350 °C, while the recombination of the formed radicals leads to guaiacyl and syringyl compounds [67]; aryl-ether bonds cleavage [68]
	345			Conversion of phenols into pyrocatechols [65]; conversion of short substituents of the benzene rings [66]
	≥400			Rearrangement of backbone, carbonization
LBL–M	149	13.4	52.4	Water evaporation, EtO elimination
	338	18.6		Condensation and elimination of the hydroxyl groups; decomposition of the aminopropyl radical; conversion of phenols into pyrocatechols (demethylation of the dimethothoxy groups) [65]
	464	16.0		Conversion of short substituents of the benzene rings [66], rearrangement of backbone, carbonization
CFBL–M	139	12.5	48.7	Water evaporation, EtO elimination
	333	15.7		Condensation and elimination of the hydroxyl groups; decomposition of the aminopropyl radical; conversion of phenols into pyrocatechols (demethylation of the dimethothoxy-groups) [65]
	422	18.9		Conversion of short substituents of the benzene rings [66], rearrangement of backbone, carbonization
LBL–Silica	55	0.7	45	Moisture evaporation
	172	4.2		Water evaporation, EtO elimination
	290	12.0		Onset of lignin decomposition
	416	25		Conversion of short substituents of the benzene rings [66], rearrangement of backbone, carbonization, hydroxyl radical elimination from silica
CFBL–Silica	50	0.5	46	Moisture evaporation
	159	12		Water evaporation, EtO elimination
	290			Onset of lignin decomposition
	414			Conversion of short substituents of the benzene rings [66], rearrangement of backbone, carbonization, hydroxyl radical elimination from silica
Silica/W	78	9.3	15.6	Physically adsorbed water evaporation
	260	15.4		Condensation of the silica hydroxyl groups
Silica/D:W	73	5.8	17.1	Dioxan evaporation
	213	13.1		Condensation of the silica hydroxyl groups

Note: M.—Material; LBL–M—LBL-Mannich; CFBL–M—CFBL-Mannich; Silica/W—Silica synthesized in aqueous media; Silica/D:W—Silica synthesized in dioxane/water media.

## Supplementary Materials

The following are available online at http://www.mdpi.com/2079-4991/8/11/950/s1, Figure S1: ^31^P-NMR spectra of the CFBL and LBL; Figure S2: FTIR spectra of the LBL-Mannich and CFBL-Mannich samples before and after precipitation and washing by diethyl ether; Figure S3: FTIR spectra (in range 4000–600 cm^−1^) of the materials based on (a) LBL and (b) CFBL: initial lignin; lignin modified by the Mannich reaction; lignin-silica hybrids and synthesized pure silica; Figure S4: TG- and DTG-curves of thermal decomposition in N_2_ atmosphere for initial and modified lignins, hybrid composites and silica; Figure S5: TG- and DTG-curves of thermal decomposition in O_2_ atmosphere for initial and modified lignins, hybrids and silica; Figure S6: Adsorption of methylene blue dye; Table S1: Characteristics of thermal decomposition in O_2_ atmosphere of studied materials.
