# Supplementary material for: Peculiarities of Synthesis and Properties of Lignin–Silica Nanocomposites Prepared by Sol-Gel Method
Source: Nanomaterials (Basel). 2018 Nov 18;8(11):950. doi: 10.3390/nano8110950 (PMC6267032; doi:10.3390/nano8110950)
Supplement: Supplementary file 1 [file nanomaterials-08-00950-s001.pdf]

Supporting information

Peculiarities of Synthesis and Properties of Lignin-Silica Nanocomposites Prepared by Sol-Gel Method

Tetyana M. Budnyak [1,2,*], Selda Aminzadeh [3], Ievgen V. Pylypchuk [4], Anastasia V. Riazanova [3], Valentin A. Tertykh [2], Mikael E. Lindström [1] and Olena Sevastyanova [1,3,*]

[1] Division of Wood Chemistry and Pulp Technology, Department of Fiber and Polymer Technology, KTH Royal Institute of Technology, Teknikringen 56-58, SE-100 44 Stockholm, Sweden; tetyanabudnyak@yahoo.com (T.M.B.); mil@kth.se (M.E.L.); olena@kth.se (O.S.)

[2] Chuiko Institute of Surface Chemistry, National Academy of Sciences of Ukraine, 17 General Naumov Str., 03164 Kyiv, Ukraine; tertykh@yahoo.com

[3] Wallenberg Wood Science Center (WWSC), Department of Fiber and Polymer Technology, KTH Royal Institute of Technology, Teknikringen 56-58, SE-100 44 Stockholm, Sweden; seldaa@kth.se (S.A.); anaria@kth.se (A.V.R.)

[4] Department of Molecular Sciences, Swedish University of Agricultural Sciences (SLU), Allmas alle 5, SE-750 07 Uppsala, Sweden; ievgenpylypchuk@gmail.com

* Correspondence: tetyanabudnyak@yahoo.com (T.M.B.); olena@kth.se (O.S.); Tel.: +46-8-790-8067 (O.S.)

Received: date; Accepted: date; Published: date

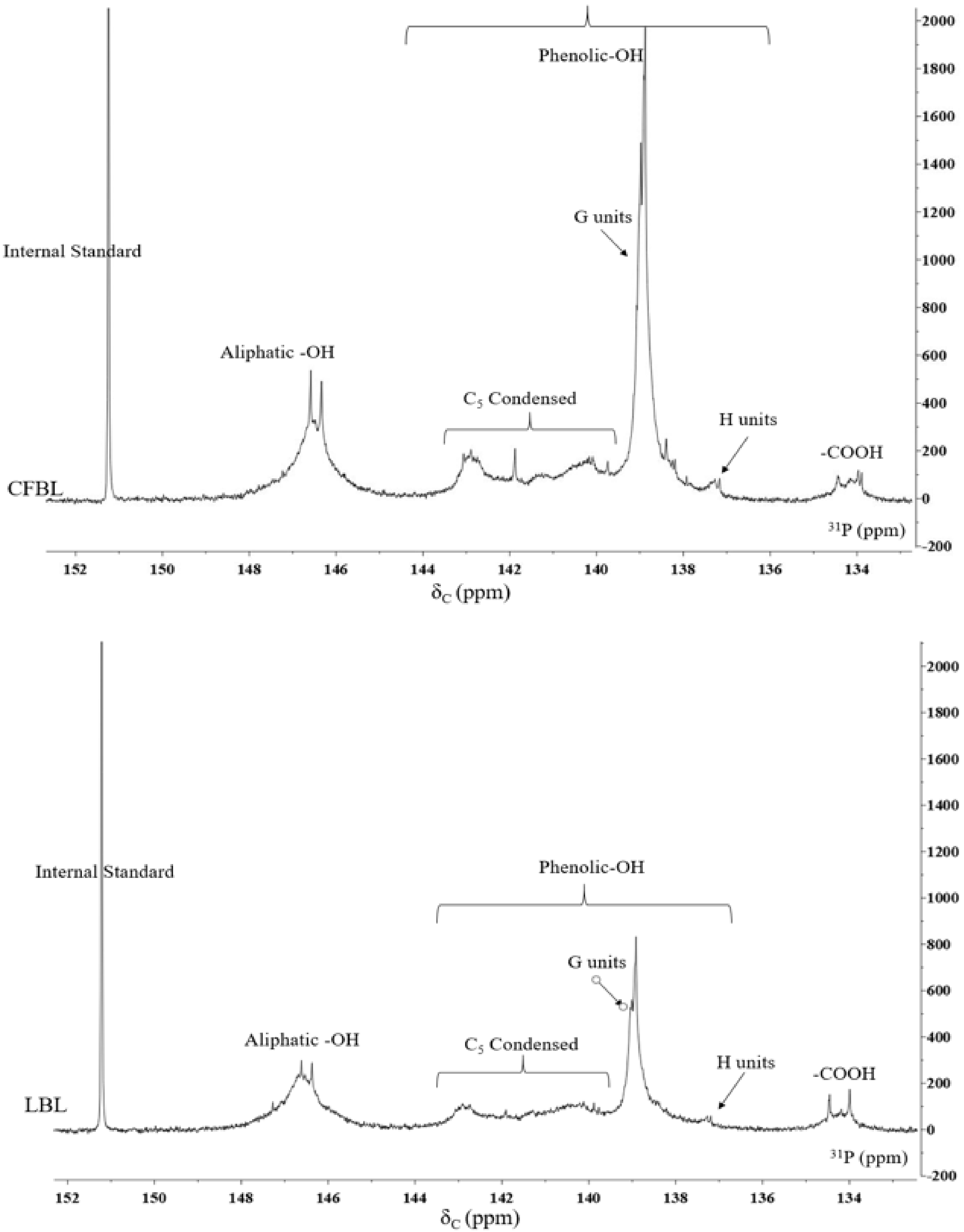

Figure S1. ^{31}P-NMR spectra of the CFBL and LBL.

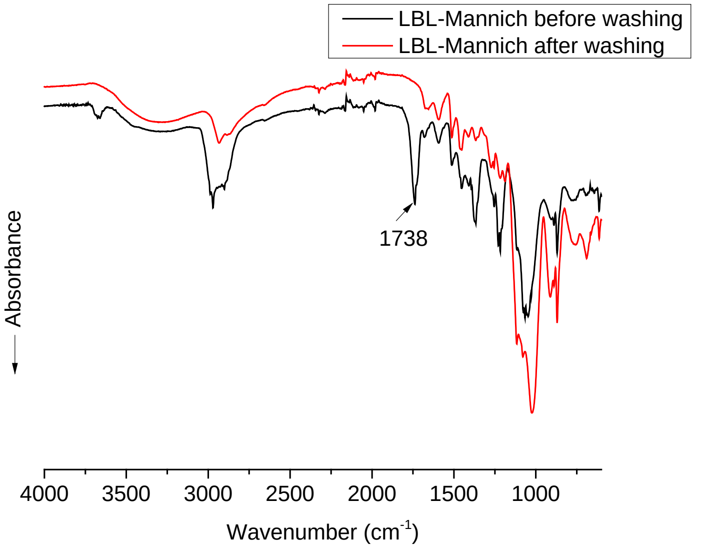

a

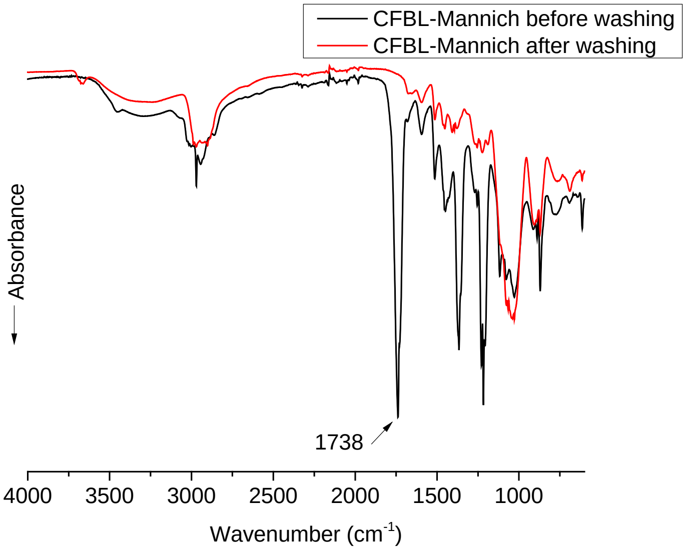

b

Figure S2. FTIR spectra of the LBL-Mannich (a) and CFBL-Mannich (b) samples before and after precipitation and washing by diethyl ether.

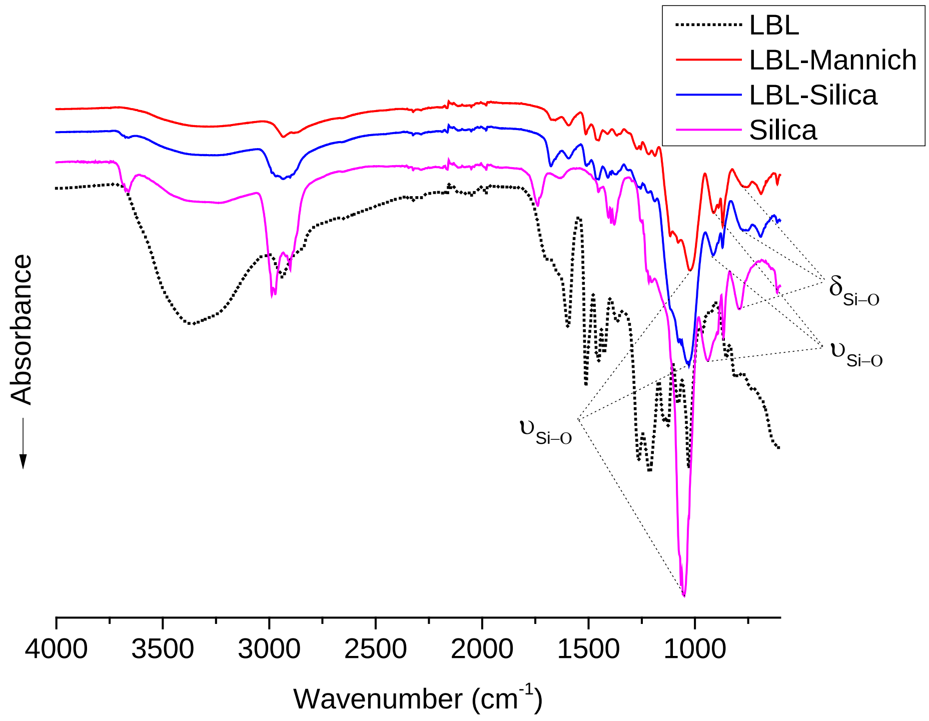

a

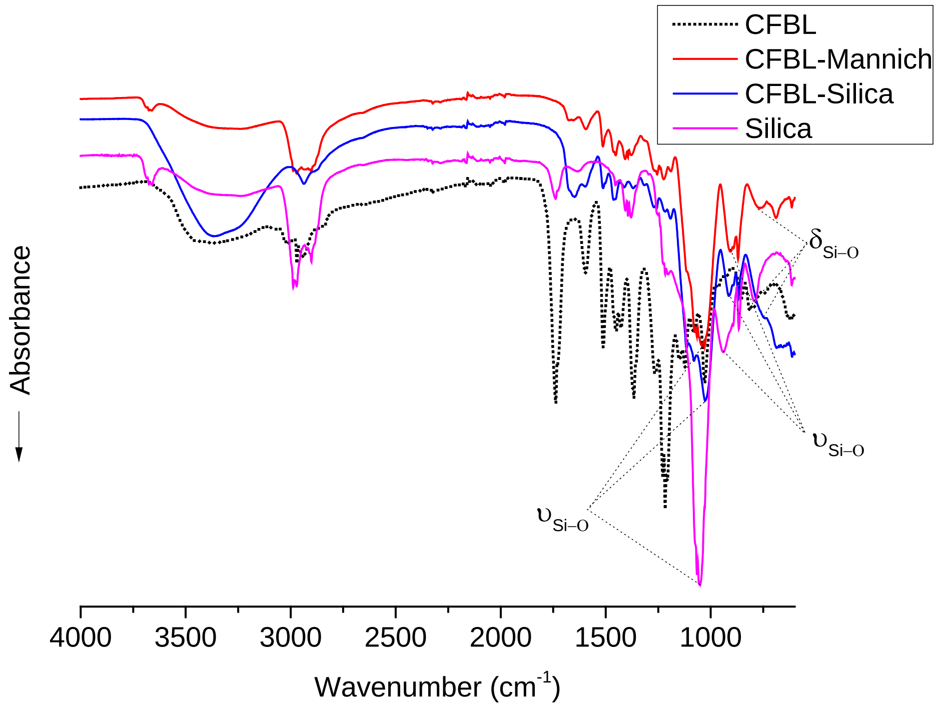

b

Figure S3. FTIR spectra (in range 4000–600 cm^{-1}) of the materials based on LBL (**a**) and CFBL (**b**): initial lignin; lignin modified by the Mannich reaction; lignin–silica hybrids and synthesized pure silica.

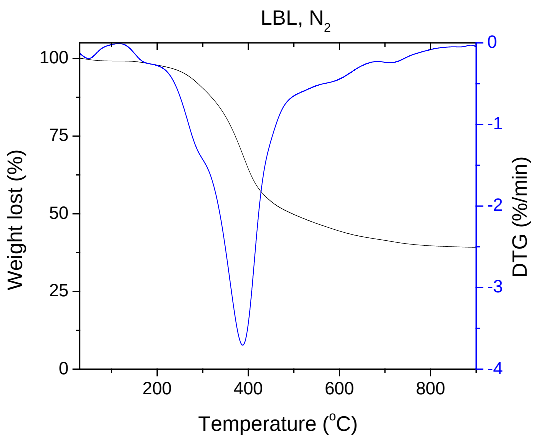
LBL, N_2
Weight lost (%)
DTG (%/min)
Temperature (°C)

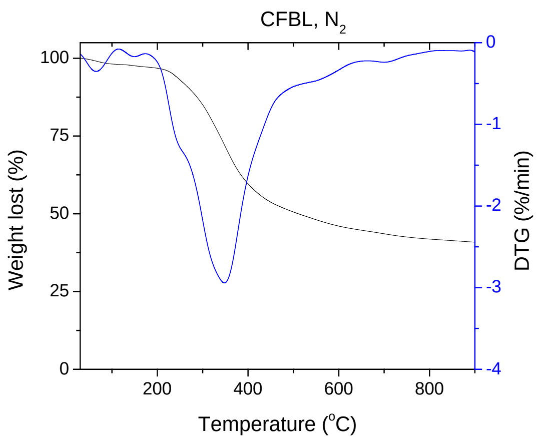
CFBL, N_2
Weight lost (%)
DTG (%/min)
Temperature (°C)

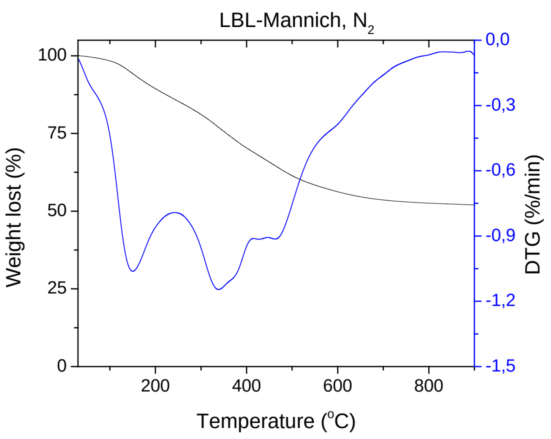
LBL-Mannich, N_2
Weight lost (%)
DTG (%/min)
Temperature (°C)

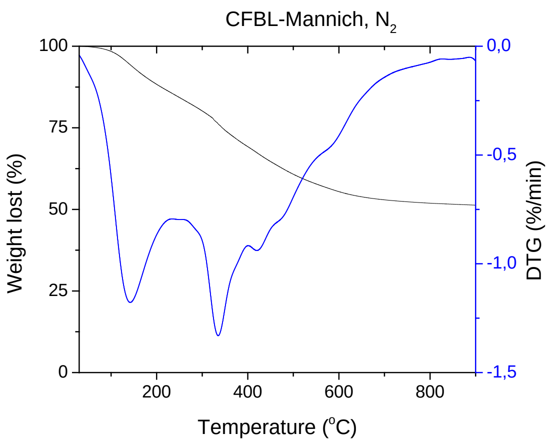
CFBL-Mannich, N_2
Weight lost (%)
DTG (%/min)
Temperature (°C)

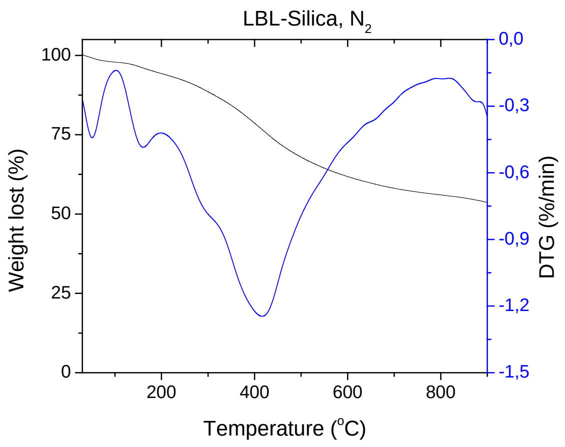
LBL-Silica, N_2
Weight lost (%)
DTG (%/min)
Temperature (°C)

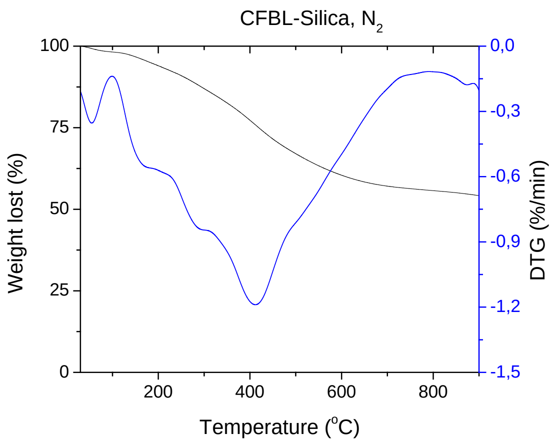
CFBL-Silica, N_2
Weight lost (%)
DTG (%/min)
Temperature (°C)

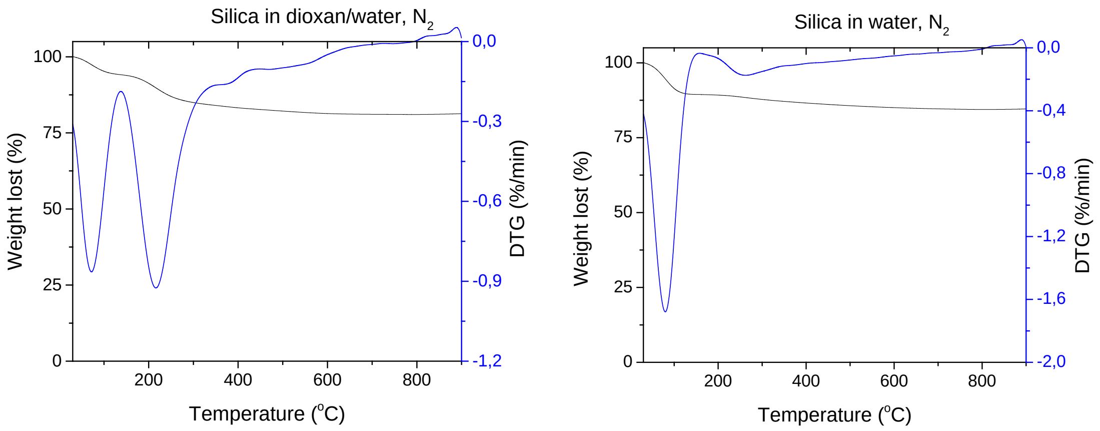

Figure S4. TG and DTG curves of thermal decomposition in N_2 atmosphere for initial and modified lignins, hybrid composites, and silica.

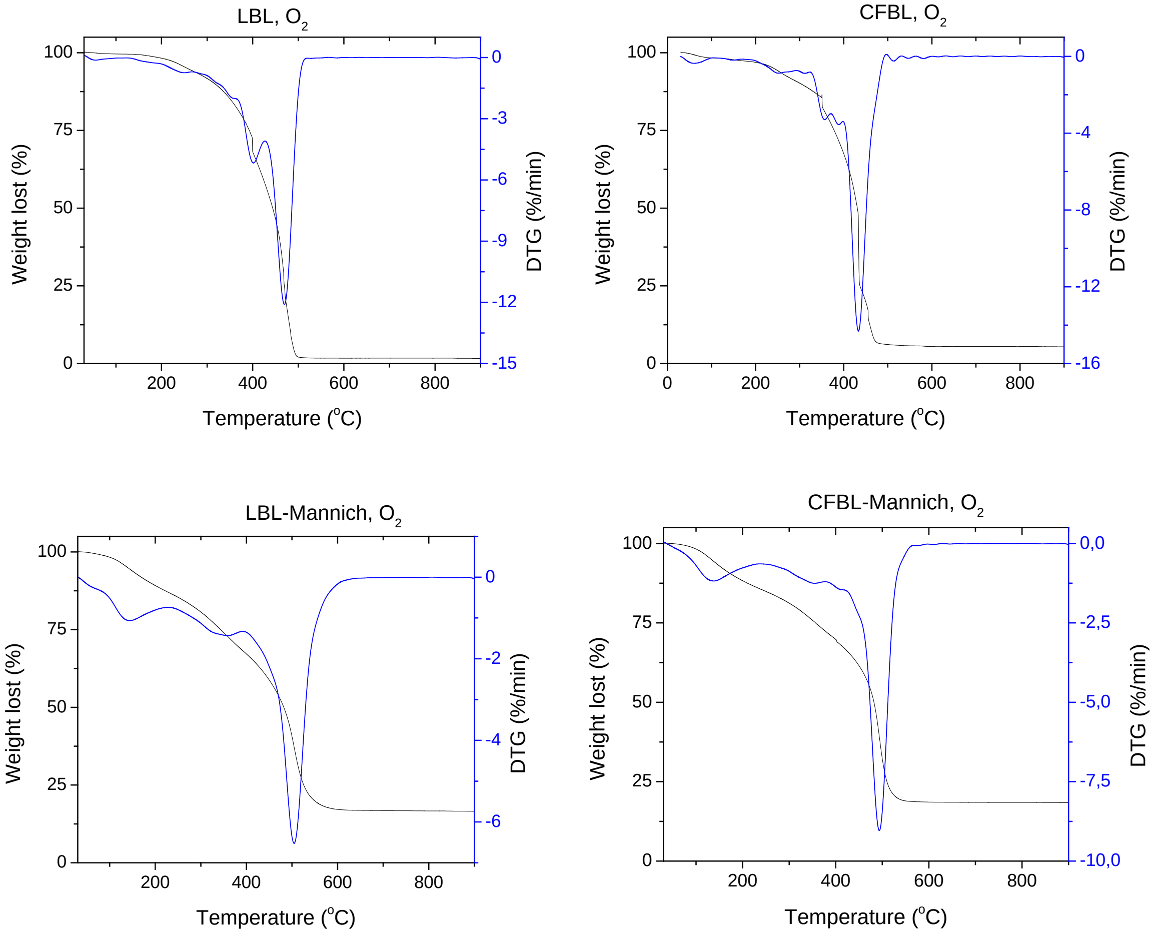

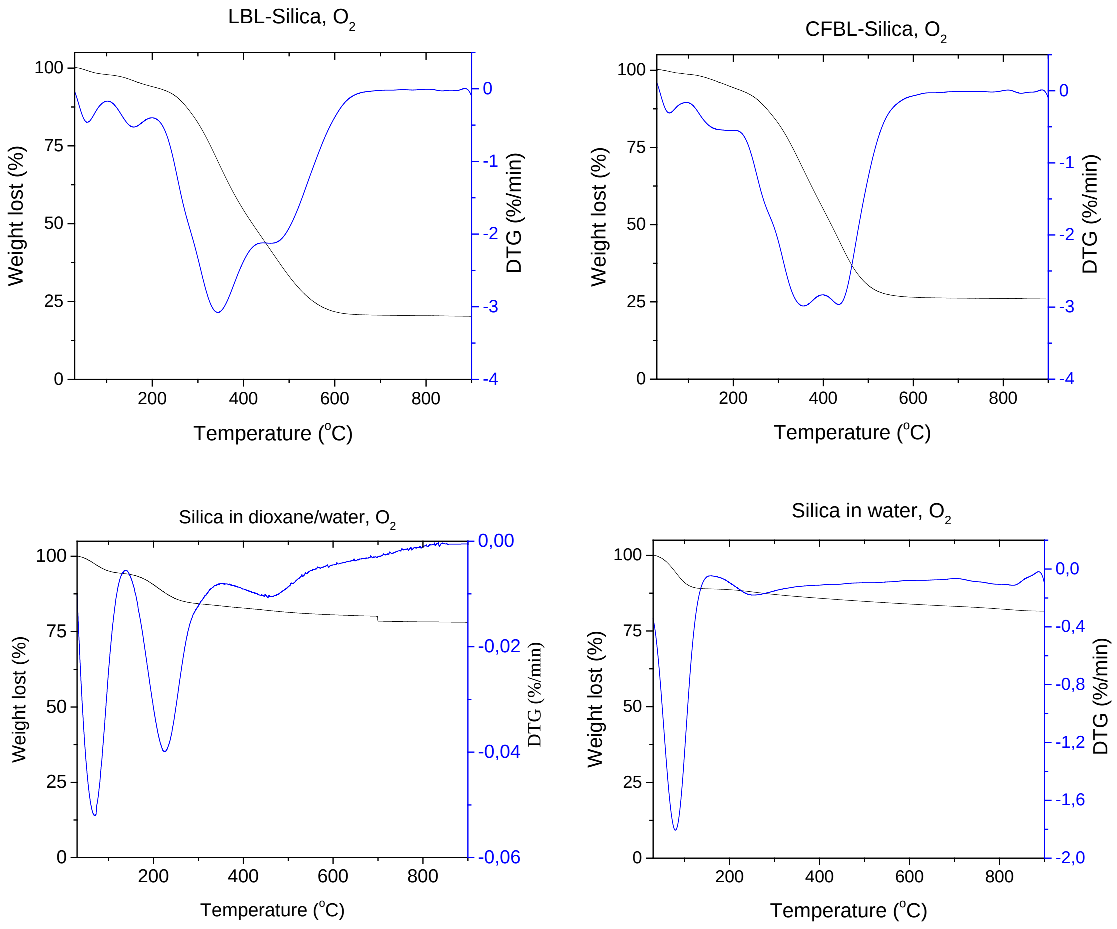

Figure S5. TG and DTG curves of thermal decomposition in O_2 atmosphere for initial and modified lignins, hybrids, and silica.

Table S1. Characteristics of thermal decomposition of studied materials in O_2 atmosphere.

Material	T_{max}, °C (DTG)	Δm, % (TG)	$Δm_{total}$, % (TG)	Process
LBL	56	0.3	97.77	Moisture evaporation
	250	6.4		Lignin decomposition onset
	401	16.3		Lignin decomposition
	468	61.6		Lignin combustion
CFBL	58	0.7	94.5	Moisture evaporation
	247	6		Lignin decomposition onset
	383	27		Lignin decomposition
	435	63.4		Lignin combustion
LBL–Mannich	54	0.5	83.4	Moisture evaporation
	142	5.2		Water and ethoxyl radical elimination
	347	26.1		Decomposition of the aminopropyl radical
	504	61.2		Lignin decomposition and combustion
CFBL–Mannich	138	13.3	82.3	Ethoxyl radical elimination
	354	19.7		Decomposition of the aminopropyl radical
	493	49.2		Lignin decomposition and combustion
LBL–Silica	59	1.4	77	Moisture evaporation
	158	5.1		Water and ethoxyl radical elimination
	344	45.9		Lignin and aminopropyl radical decomposition
	470	29.4		Lignin combustion
CFBL–Silica	58	1.4	73	Moisture evaporation
	158	5.1		Water and ethoxyl radical elimination
	353	38.44		Lignin and aminopropyl radical decomposition
	435	28.3		Lignin combustion

Silica in water	67<br>210	11<br>2	22.1	Physically adsorbed water evaporation;<br>condensation of the silica hydroxyl groups
Silica in dioxane/water	80<br>248	5.4<br>10.75	18.3	Dioxan evaporation;<br>condensation of the silica hydroxyl groups

Adsorption of methylene blue dye

The synthesized hybrid composites were tested as sorbents for the removal of methylene blue dye from aqueous solutions. This study was conducted to evaluate the dependence of the sorption properties of the synthesized hybrid materials on the structure of the lignin component and to compare the sorption properties of the initial LignoBoost and CleanFlowBlack lignins.

The adsorption capacity of the hybrids, initial lignins, and silica were estimated from the adsorption isotherms of methylene blue dye in a neutral medium. The equilibrium studies were conducted over 48 h for all systems. The obtained results were compared and are shown in a diagram in Figure 10. As seen from Figure 10, the adsorption capacity of the hybrid composites is higher than that of the original lignins. In the case of LBL (adsorption capacity = 31.2 mg·g^{-1}), the increase reached 30% (adsorption capacity of LBL–silica = 41.6 mg·g^{-1}), and in the case of CFBL (adsorption capacity of CFBL–silica = 32.3 mg·g^{-1}), the adsorption capacity almost doubled (adsorption capacity = 58.9 mg·g^{-1}). It could be concluded that the increase in the adsorption capacity is presumably related to an increase in the organophilic properties of the hybrid composites. The highest sorption of the studied dye was observed for the CFBL–silica composite. The lignin in this composite has a less condensed structure, a lower molecular weight, and a larger number of functional groups. Based on the higher reactivity, the lignin was probably more homogeneously distributed on the surface of the hybrid composite, which resulted in better accessibility of the functional groups that participated in the sorption process.

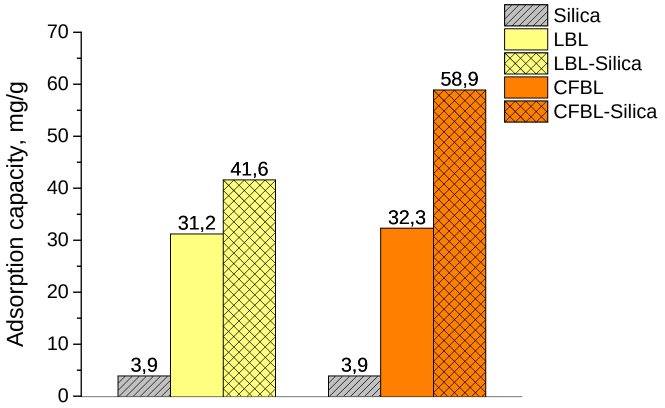

Figure S6. Adsorption capacities of the synthesized silica, initial CFBL and LBL, and LBL–silica and CFBL–silica composites for methylene blue dye in a neutral medium.
